# Lewy Body-like Pathology and Loss of Dopaminergic Neurons in Midbrain Organoids Derived from Familial Parkinson’s Disease Patient

**DOI:** 10.3390/cells12040625

**Published:** 2023-02-15

**Authors:** Andrea Becerra-Calixto, Abhisek Mukherjee, Santiago Ramirez, Sofia Sepulveda, Tirthankar Sinha, Rabab Al-Lahham, Nicole De Gregorio, Camila Gherardelli, Claudio Soto

**Affiliations:** Mitchell Center for Alzheimer’s Disease and Related Brain Disorders, Department of Neurology, McGovern Medical School, University of Texas Health Science Center at Houston, Houston, TX 77030, USA

**Keywords:** human midbrain-like organoids, Parkinson’s disease, 3D models, Lewy body disease

## Abstract

Progressive accumulation of α-Synuclein (αSyn) in Lewy bodies (LBs) and loss of dopaminergic (DA) neurons are the hallmark pathological features of Parkinson’s disease (PD). Although currently available in vitro and in vivo models have provided crucial information about PD pathogenesis, the mechanistic link between the progressive accumulation of αSyn into LBs and the loss of DA neurons is still unclear. To address this, it is critical to model LB formation and DA neuron loss, the two key neuropathological aspects of PD, in a relevant in vitro system. In this study, we developed a human midbrain-like organoid (hMBO) model of PD. We demonstrated that hMBOs generated from induced pluripotent stem cells (hiPSCs), derived from a familial PD (fPD) patient carrying αSyn gene (*SNCA*) triplication accumulate pathological αSyn over time. These cytoplasmic inclusions spatially and morphologically resembled diverse stages of LB formation and were composed of key markers of LBs. Importantly, the progressive accumulation of pathological αSyn was paralleled by the loss of DA neurons and elevated apoptosis. The model developed in this study will complement the existing in vitro models of PD and will provide a unique platform to study the spatiotemporal events governing LB formation and their relation with neurodegeneration. Furthermore, this model will also be beneficial for in vitro screening and the development of therapeutic compounds.

## 1. Introduction

Parkinson’s disease (PD) is the most common synucleinopathy and is also considered to be the second most prevalent neurodegenerative disorder, after Alzheimer’s disease (AD) [1]. The main pathological features of PD involve the presence of αSyn aggregates in the form of intracellular inclusions, referred to as Lewy bodies (LBs) and Lewy neurites (LNs) [2]. The presence of these inclusions has been linked to the loss of DA neurons, especially in the substantia nigra (SN) of the midbrain [2,3].

Currently, most of the studies of αSyn misfolding, aggregation, propagation and neurotoxicity employ diverse in vivo models (transgenic, wild-type mice and rats) [4,5,6,7]. However, unlike humans, rodents do not develop PD spontaneously and their cellular, molecular and gene expression profiles are also vastly different [8]. To address this issue, human-immortalized cell line-based models of PD were developed [9,10,11]. We and others recently generated human-induced pluripotent stem cells (hiPSCs)-derived DA neurons to study αSyn pathology [12,13]. However, these in vitro models lack the three-dimensional architecture and multicellular heterogeneity of the human brain [14,15], which imposes a limitation on the translational potential of these models.

hiPSCs-derived 3D brain cell culture models provide a unique opportunity to examine complex cellular interactions governed by the human genome [16,17,18,19]. Specifically, cerebral organoids and cortical spheroids [20,21] recapitulate features of pre- and post-natal human brain in vitro, including the generation, proliferation and differentiation of neural progenitors into neurons and glial cells, and their maturation, together with their complex interactions in 3D [21,22]. Importantly, organoids can be generated to mimic different brain regions, such as the forebrain [23], midbrain [24,25,26] and hindbrain [27], therefore providing platforms to study the neuropathology of a specific region [28,29,30]. Importantly, we and others have recently modeled key pathological features of neurodegenerative diseases, including AD and traumatic brain injury in cerebral organoids [29,31,32,33,34,35].

Recently, multiple groups have attempted modeling PD-like pathology in hMBOs carrying different PD-associated mutations [24,25,26,30]. However, until now there has been a lack of a model that reproduces LB formation and DA neuron loss, without the need of genetic manipulation or exposure to toxin. In this study, using hiPSCs from a fPD patient with *SNCA* gene triplication, we have successfully generated hMBOs bearing key pathological features of PD, including LB-like inclusions and the loss of DA neurons. We demonstrated that in these PD-hMBOs, αSyn spontaneously accumulated into inclusions without the need for any genetic manipulation or chemical exposure. These organoids spatially and morphologically recapitulate critical aspects of LBs and are composed of key markers of LBs. Our study provides a valuable platform to investigate the pathophysiology of LBs in a 3D model, composed of a variety of human brain cells, organized in a brain-like manner.

## 2. Materials and Methods

### 2.1. hiPSCs Information

The hiPSCs lines used and the experiments performed with hMBOs were approved by Stem Cell Research Oversight Committees at UT Health Houston. For this study, we used hiPSC line Edi044-A, derived from an 80-year-old healthy female subject. This line was acquired from the Cedars Sinai stem cell bank. We also used a familial PD hiPSC line (NDS00201) obtained from a 55-year-old female subject with *SNCA* gene triplication, who was diagnosed with PD at the age of 52. This line was acquired from the National Institute of Neurological Disorders and Stroke (NINDS) Human Cell and Data Repository.

### 2.2. hiPSCs Culture Maintenance

hIPSCs were cultured in 6-wells plates coated with hESC-Qualified Matrix, LDEV-free, Matrigel^®^ (Corning^®^ 354277, Corning, NY, USA) using mTeSRTM Plus medium (StemCellTM Technologies 100-0276, Vancouver, BC, Canada). To ensure the stabilization and homogeneity of the colonies before generating the 3D cultures, both types of cells were passaged three times. For each passage, the cells were dissociated 1:6 using ReLeSRTM (StemCellTM Technologies). Mycoplasma tests (Millipore Sigma MP0035-1KT, St. Luis, MO, USA) were performed monthly for both hiPSCs and hMBOs.

### 2.3. Brain Sections from LBD Patient

Formalin-fixed/paraffin-embedded midbrain sections from pathologically confirmed LBD (Lewy body disease) patients were kindly provided by our colleagues at the Mayo Clinic: Dr. Wolfgang Singer, Dr. Ann Schmeichel and Dr. Phillip Low. Research on human samples was performed following The Code of Ethics of the World Medical Association (Declaration of Helsinki). Samples were handled according to the universal precautions for working with human samples and as directed by the Institutional Review Board of the University of Texas Health Science Center at Houston.

### 2.4. Generation of hMBOs

**Figure 1:**
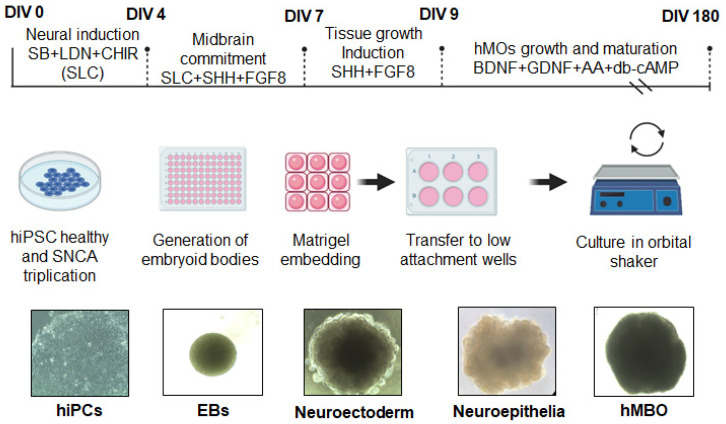
Schematic description of the protocol to generate hMBOs. Schematic diagram illustrating the protocol to generate hMBOs and representative brightfield images of hMBOs at different stages of differentiation. The scale bar for image DIV 0 is 100µm, DIV4 and DIV7 is 260 µm and for images, DIV 11 and DIV30 is 520 µm.

hMBOs were generated as schematically represented in Figure 1, based on a previously published method [26]. Briefly, hiPSC colonies were dissociated into a single-cell suspension. A total of 15,000 hiPSCs/well were used to form embryoid bodies (EBs) in a 96-well plate. The EBs were cultured in a neural induction medium DMEM F-12 and neurobasal medium, 1:1 respectively N2 1X, B27 1X, Glutamax 1X, MEM NEAA 1X, beta-mercaptoethanol 55 µM (12 (Thermoscientific^®^ Waltham, MA, USA), heparin 1µg/mL (StemCellTM Technologies), SB431542 100 mM, LDN 2 mM, CHIR 10 mM, supplemented with 10 µM ROCK inhibitor Y-27632 (Tocris^®^ Bristol, UK). The ROCK inhibitor was added for the first 48 h, and the neuronal induction medium was changed on day 2. On day 4, the EBs were supplemented with midbrain patterning factors, 100 ng/mL Sonic Hedgehog, C25II (SHH-C25II) 25 µg/mL and fibroblast growth factor 8 (FGF8) (R&D Systems, Minneapolis, MN, USA), for 3 days. Subsequently, the EBs were embedded in 30 μL of reduced growth factor Matrigel (Corning^®^ Matrigel^®^ 354230, NY, USA) and cultured in tissue growth and induction medium supplemented with 100 ng/mL SHH-C25II and FGF8 and transferred to ultra-low attachment 6-well plates (Corning, Costar^®^ 3471 NY, USA). At day 7, neuroectodermal spheroids were embedded in Matrigel^®^ and transferred to low attachment wells using a medium 1:100 N2 supplement (Invitrogen, Waltham, MA, USA), 1:50 B27 without vitamin A (Invitrogen), 1% GlutaMAX (Invitrogen), 1% and 0.1% β-mercaptoethanol (Invitrogen) supplemented with 2.5 mg/mL insulin (Sigma-Aldrich, St. Luis, MO, USA), 200 ng/mL laminin (Sigma-Aldrich), 100 ng/mL SHH-C25II and 100 ng/mL FGF8 (R&D Systems) for 48 h. Finally, on day 9, hMLOs were transferred into ultra-low attachment 6-well plates (Costar) by pipetting using a cut 1000 μL tip; the plates contained organoid differentiation medium which consisted of neurobasal medium 1:100 N2 supplement (Invitrogen), 1% GlutaMAX (Invitrogen), 1% minimum essential media with non-essential amino acid (Invitrogen) and 0.1% β-mercaptoethanol supplemented with 10 ng/mL BDNF (R&D 248-GMP NE Minneapolis, MN, USA), 10 ng/mL GDNF (R&D 21 2-GMP NE Minneapolis, USA), 100 μM ascorbic acid (Millipore- SigmaTM A92902 St. Luis, MO, USA) and 125 µM dybutytil-cAMP (Tocris^®^ 1141, Bristol, UK), antibiotics (100 U/penicillin G and 100 μg/mL streptomycin), and the medium was consistently shaken on an orbital shaker at 70 rpm. The medium was replenished every 3–4 days (Figure 1).

### 2.5. Fixation and Immunohistochemistry (IHC)

hMBOs were fixed in 4% paraformaldehyde for 48 h. The dehydration process was performed using graded ethanol and xylenes followed by final embedding in paraffin. Paraffin blocks were cut using a microtome at a thickness of 12 µm. For antigen retrieval, the slides were rehydrated and exposed to citrate buffer (ab93678 Abcam, Cambridge, MA, USA). Endogenous peroxidase activity was blocked via incubation in 3% H_2_O_2_/10% methanol in PBS for 20 min on a rocking platform. Non-specific binding was blocked in 3% donkey serum/PBS containing 0.3% TritonX-100 for 30 min, followed by incubation overnight in a humidity chamber with the primary antibodies, anti-phospho-Ser129-α-synuclein (pS129) (1:200, Cat. Ab51253, Abcam, Cambridge, MA, USA) and anti p62 antibody (1:200, Cat ab280086C, Abcam, Cambridge, MA, USA), prepared in 3% donkey serum/PBS-0.3% TritonX-100 at room temperature. The next day, the sections were washed in PBS and then incubated for 1.5 h with an HRP-linked secondary sheep anti-rabbit (1:1000 Cat. A0545 anti-rabbit peroxidase, Sigma Aldrich, St. Luis, MO, USA) and anti-mouse antibodies (1:1000 Cat. A5906 anti-mouse peroxidase, Sigma Aldrich, St Luis MO USA) at room temperature. After washing with PBS, the peroxidase reaction was visualized using 3, 3′-diaminobenzidine (DAB) as a chromogen (Vector Laboratories, Burlingame, CA, USA) following the manufacturer’s instructions. Sections were then counterstained with hematoxylin for 40 s at room temperature and washed in water. Finally, sections were dehydrated in graded ethanol, cleared in xylene, and mounted using DPX Mounting Medium (Electron Microscopy Sciences, Hatfield, PA, USA). LBD midbrain sections were treated similarly and immunostained with the same antibodies. IHC Images were taken using the color camera of the Leica Stellaris^®^ confocal microscope (Leica Microsystems, Buffalo Grove, IL, USA); the photographs were acquired using LAS-X software (Leica Microsystems, Buffalo Grove, IL, USA).

### 2.6. Immunofluorescence (IF)

After deparaffinization and hydration, sections were blocked with 3% donkey serum/PBS containing 0.3% TritonX-100 for 1 h at room temperature. Sections were then incubated with primary antibodies overnight (see antibodies list, Table S1) at room temperature. Respective secondary Anti-Mouse Alexa Fluor-594 (1:1000, Invitrogen™ A32744, Waltham, MA, USA) or Anti-Rabbit Alexa Fluor-488 (1:1000 Invitrogen™ A32790 Waltham, MA, USA) antibodies were incubated for one hour at room temperature. All sections were counterstained with (4′,6-diamidino-2-phenylindole) DAPI (Millipore Sigma 10236276001, St. Luis, MO, USA) and mounted with FluorSaveTM (Millipore- Sigma 345789, St. Luis, MO, USA), protected from the light. Images were taken using a confocal and epifluorescence microscope, Leica Stellaris^®^ (Leica Microsystems, Buffalo Grove, IL); photographs were digitalized using LAS-X software (Leica Microsystems, Buffalo Grove, IL) and imported into ImageJ 1.45 s software (NIH) for analysis. ROI for analysis was defined via the presence of MAP2 (1:400, BD Pharmingen™ 556320, Franklin Lakes, NJ, USA)-immunoreactive areas of the organoid. Immunostainings for cleaved Caspase 3 (c-Casp3) (1:500 Abcam™ ab2302) were evaluated in 20X microphotographs, and the ratio of cCasp3-positive cells to DAPI-positive cells was quantified. Areas immunoreactive for TH and pS129 were quantified and expressed relative to the DAPI area and total αSyn area, respectively. Images were analyzed blindly.

### 2.7. Statistical Analysis

Analysis was performed using 3 images from 3 sections of each organoid per time point in each group. Data were analyzed using a two-way ANOVA to evaluate the effect of two variables [donors’ *SNCA* copy number (normal and triplication) and the time in culture (120 and 180 days in vitro) for the following analyses: αphosphorylation, as assessed using the pS129/αSyn ratio, cleaved caspase 3 levels, and TH/DAPI ratio. We used Tukey’s test for pairwise post hoc comparisons. Significant differences were considered with *p* < 0.05.

### 2.8. Ethical Approvals

Experiments conducted with human iPSCs and employing human brain sections for staining were approved by the Institution Stem Cell Committee and Institutional Review Board of the University of Texas Health Science Center at Houston.

## 3. Results

### 3.1. Generation hMBOs from Healthy and fPD hiPSC Lines

**Figure 2:**
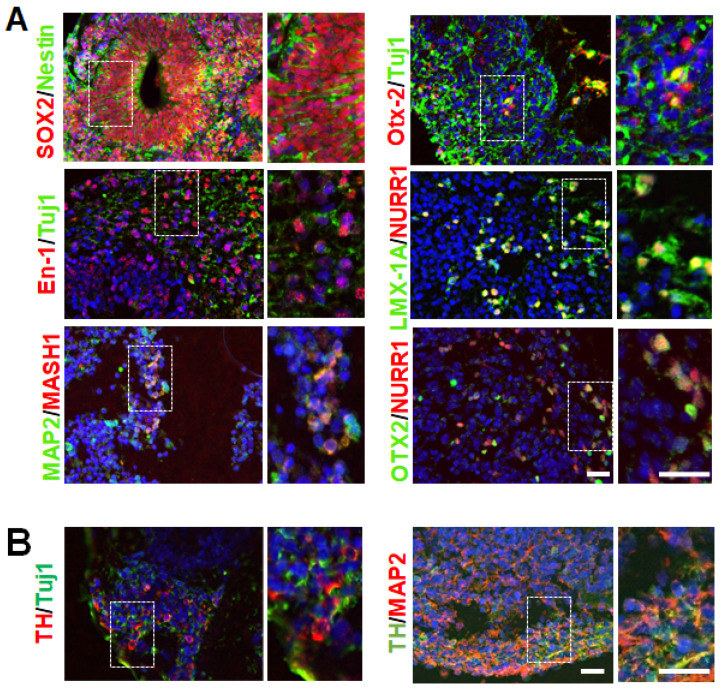
Generation and characterization of hMBOs from hiPSCs. (**A**). Representative immunostaining images of hMBOs characterization at 30 DIV using neuroepithelial markers (SOX2, Nestin), dorsal midbrain and DA progenitor markers (Otx2, En-1, LMX-1A, NURR1 and MASH1). (**B**). Representative immunostaining images using DA neuron marker TH with a pan-neuronal marker Tuj1 and postmitotic neuronal marker MAP2. All slides were counterstained with DAPI (blue) to mark nucleus. Dotted squares represent the magnified areas shown on the right side of the main image. The scale bar is 50 µm.

DA neurons of the substantia nigra pars compacta in the midbrain are the primary cells affected in PD [2]. In order to generate and characterize hMBOs containing DA neurons, we used an hiPSC line from an 80-year-old healthy female and a 55-year-old female patient with fPD carrying *SNCA* gene triplication. The hiPSCs colonies displayed compact morphology with defined borders and expressed typical pluripotency markers (Figure 1 and Figure S1). We generated hMBOs from these lines based on a previously published method [26] (Figure 1). Next, we characterized the hMBOs derived from the healthy donor using immunofluorescence (IF) (Figure 2) to confirm the midbrain identity via the presence of midbrain progenitor cells. After 30 days in vitro (DIV), the hMBOs expressed the neuroepithelial markers SOX2 and Nestin (Figure 2A), which are essential for the proliferation and maturation of neural progenitors, as well as Mash 1, which promotes the neuronal commitment of multipotent progenitors [36,37]; the transcription factors associated with patterning of dorsal midbrain (En-1 and Otx2) [38,39]; and midbrain DA (mDA) neuron progenitor markers (Lmx1a and Nurr1) [40,41] (Figure 2A). We detected the immunoreactivity of microtubule-associated protein 2 (MAP2), a postmitotic neuronal marker, and thyroxine hydroxylase (TH), a marker of DA neurons, in the outermost layers of the hMBOs (Figure 2B).

### 3.2. αSyn Pathology in hMBOs Derived from the fPD Patient

**Figure 3:**
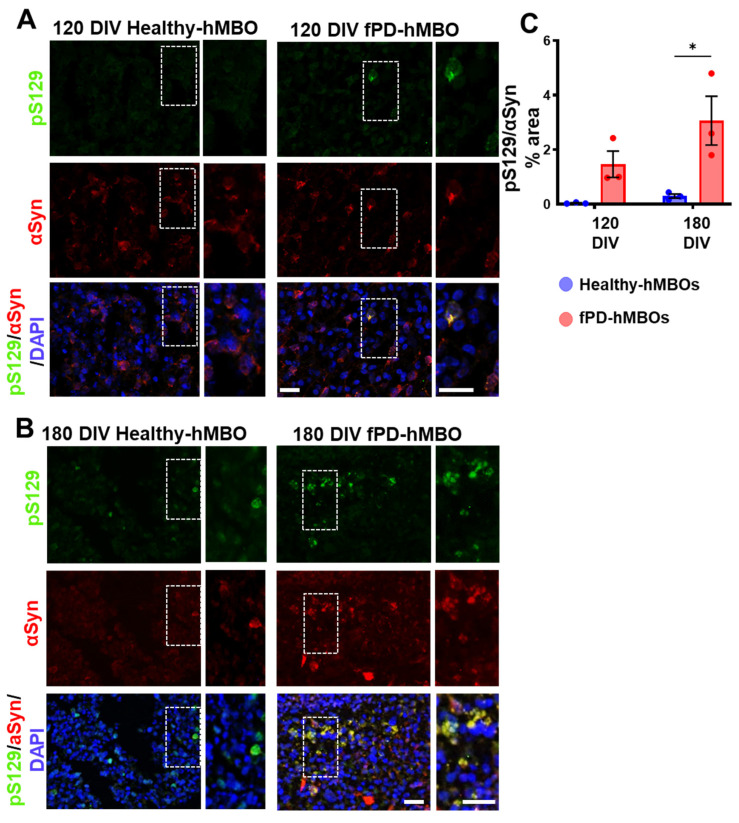
Accumulation of pathological αSyn in fPD-hMBOs. Representative images from healthy hMBOs and fPD-hMBOs, immunostained for pS129 (green) and αSyn (red), at 120 DIV (**A**) and 180 DIV (**B**). Nuclei were stained with DAPI (blue). Dotted squares represent the magnified areas shown on the right side of the main image to highlight the juxtanuclear accumulation of phosphorylated αSyn. The scale bar is 50 µm. (**C**). Immunostaining quantification at 120 and 180 DIV of pS129 normalized with total αSyn area. Values represent mean +/− standard error of mean (SEM) from *n* = 3 hMBOs per group. Three sections were analyzed from each hMBO. The results were analyzed using two-way ANOVA [*SNCA* copy number: F (1, 8) = 16.96, *p* = 0.0034; time: F (1, 8) = 3.308, *p* = 0.5318; *SNCA* copy number x time: F (1, 8) = 1.734, *p* = 0.2243)] followed by the Tukey post hoc multiple comparisons test. * *p* < 0.05.

To evaluate the spontaneous appearance and progressive accumulation of αSyn in organoids, we generated hMBOs from the fPD-hiPSC line with *SNCA* triplication (henceforth fPD-hMBO) and healthy hiPSC line (henceforth healthy hMBO). hMBO were kept in culture for 120 and 180 days. Phosphorylation of αSyn at the residue serine 129 (pS129) is a well-established marker of αSyn accumulated in LBs [42,43]. Pathological αSyn accumulation was analyzed in hMBOs using immunostaining with anti-pS129 antibody (Figure 3A [120 DIV] and B [180 DIV], top panel in green). pS129-immunostaining was barely detectable in the healthy hMBOs, both at 120 and 180 DIV. In contrast, pS129 immunostaining began to appear at 120 DIV (Figure 3A) and became more abundant as perinuclear puncta in the fPD-hMBOs at 180 DIV (Figure 3B). We also co-immunostained the sections with anti-αSyn antibody (Figure 3A [120 DIV] and B [180 DIV]). Most of the pS129 reactivity colocalized with αSyn immunostaining, indicating that these are indeed αSyn deposits positive for the LB marker pS129. Image analysis indicated that fPD-hMBOs have higher levels of pS129 immunostaining compared to the healthy-hMBOs at 120 DIV (Figure 3C). However, the difference did not reach statistical significance. Nevertheless, this difference became even more pronounced and significant at 180 DIV (Figure 3C). These results indicate that fPD-hMBOs spontaneously accumulated pathological αSyn, which tends to progressively increase in quantity.

### 3.3. LB-Like Pathology in fPD-hMBOs

**Figure 4:**
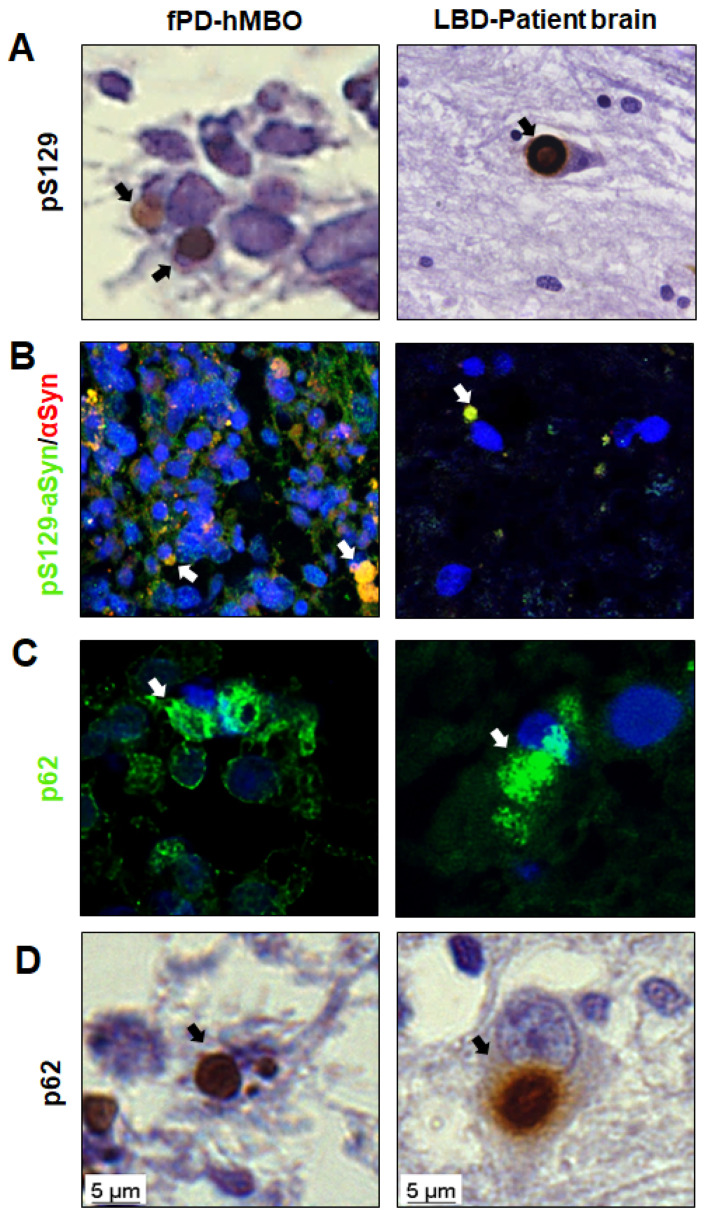
Morphology and composition of LB-like αSyn accumulates in fPD-hMBOs. (**A**). Representative immunohistochemistry (IHC) images for pS129 counterstained with hematoxylin. (**B**). Representative IF images for pS129/αSyn and DAPI. (**C**). Representative IF images for p62 and DAPI. (**D**). Representative IHC images for p62 counterstained with hematoxylin. In all panels, the left shows the results with hMBOs, and the right shows the brain of patient affected by LBD as positive control. Arrows show the LB-like deposits in fPD-hMBOs and LBs in LBD brain sections. Scale bar 5 µm.

Given the significant and progressive accumulation of pathological αSyn in fPD-hMBOs, we further investigated their morphological, spatial and compositional similarities with LBs. As a positive control, we used human brain sections from a subject with pathologically confirmed LBD. Excitingly, in fPD-hMBO sections at DIV 180, pS129 immunostained structures accumulated and were juxtaposed to the nucleus with spherical morphology and smooth edges (Figure 4A). Although not abundant, this immunostaining pattern was spatially and morphologically similar to that observed in the LBD brain section (Figure 4A. right column). Co-immunostaining with pS129 and αSyn further validated that these pS129 immunoreactive deposits are indeed composed of αSyn (Figure 4B). Nevertheless, it is important to note that we also frequently observed granular pS129 immunostaining distributed in the cytoplasm, likely resembling pale bodies observed in the early stages of LB formation [44,45]. Ubiquitin-binding protein p62 is often found in LBs and used as a LB marker [44,46]. In order to further evaluate the composition of LB-like deposits, we immunostained fPD-hMBO sections with anti-p62 antibody. LBD brain sections were used as a positive control. In both the LBD brain tissue and fPD-hMBOs, p62 immunostaining showed perinuclear inclusions of different sizes (Figure 4C). To further confirm, we also performed IHC for p62 with hematoxylin as a counterstaining. The pattern of p62 immunostaining observed in fPD-hMBOs (Figure 4D, left column) was remarkably similar to that observed in LBD brain sections (Figure 4D, right column) and consistent with the pS129 immunostaining pattern. Taken together, these data indicate that fPD-hMBOs can spontaneously develop αSyn deposits, which are spatially, and morphologically similar to different stages of LB formation and contain typical LB markers.

### 3.4. Increased Neurodegeneration in fPD-hMBOs

**Figure 5:**
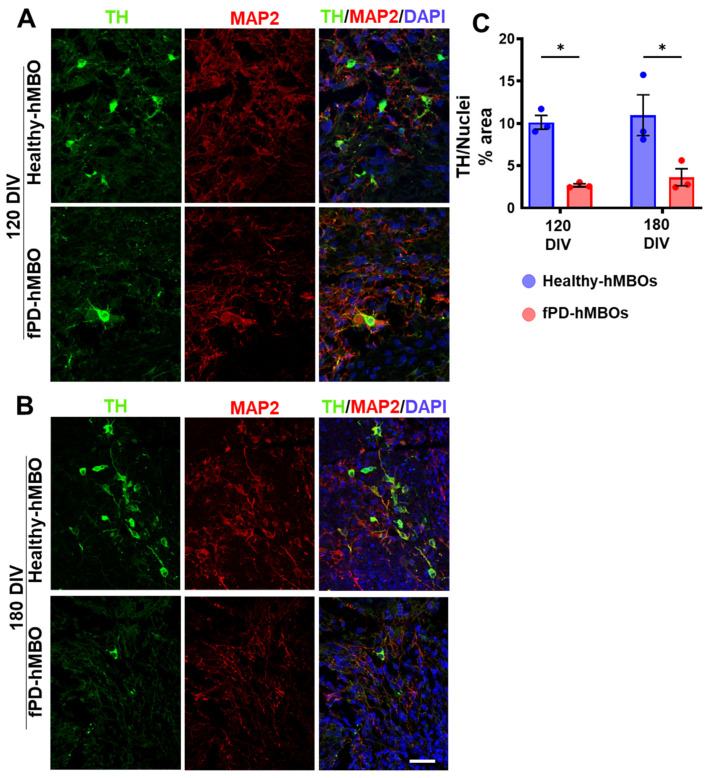
Loss of DA neurons in fPD-hMBOs. Representative images from healthy hMBOs and fPD-hMBOs, immunostained for TH (green) and MAP2 (red), at 120 DIV (**A**) and 180 DIV (**B**). Nuclei were stained with DAPI. Scale bar is 25 µm. (**C**). Quantification of TH immunostaining at 120 and 180 DIV. Values represent mean +/− standard error of mean (SEM) from *n* = 3 hMBOs per group. Three sections were analyzed from each hMBO. The results were analyzed using two-way ANOVA [*SNCA* copy number: F (1, 8) = 29.15, *p* = 0.0006; time: F (1, 8) = 0.4270, *p* = 0.5318; *SNCA* copy number × time: F (1, 8) = 0.001276, *p* = 0.9724)] followed by the Tukey post hoc multiple comparisons test. * *p* < 0.05.

Next, we investigated the degenerative consequences of αSyn accumulation. Selective loss of DA neurons is a hallmark feature of PD pathology [2,3,47]. We immunostained hMBO sections with the DA neuron marker tyrosine hydroxylase (TH) [48] (Figure 5A [120 DIV] and B [180 DIV]). Image analysis indicated that fPD-hMBOs have significantly reduced TH-positive neurons compared to healthy hMBOs, both at 120 and 180 DIV (Figure 5C). These results suggested a loss of DA neurons in hMBOs derived from fPD iPSCs.

**Figure 6:**
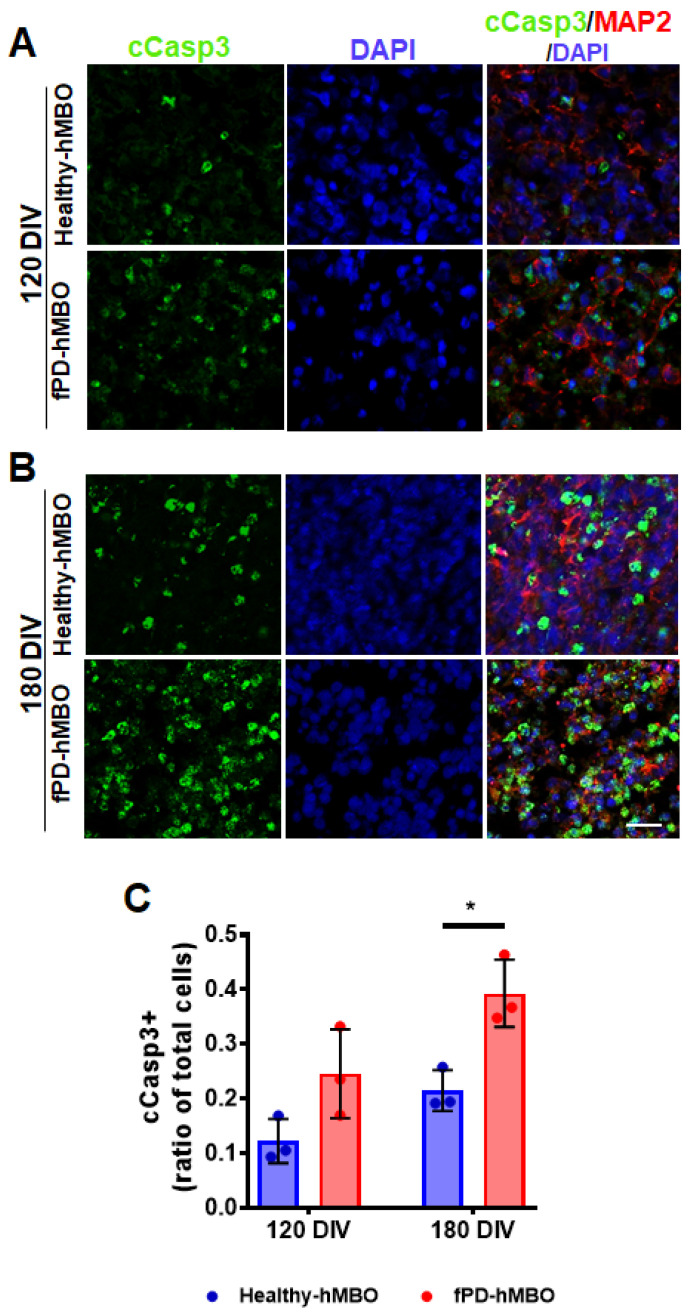
Elevated apoptosis in fPD-hMBOs. Representative images from healthy hMBOs and fPD-hMBOs, immunostained for c-Casp3 (green) and MAP2 (red), at 120 DIV (**A**) and 180 DIV (**B**). Nuclei were stained with DAPI. The scale bar is 25 µm. (**C**). Quantification of c-Casp3 immunostaining at 120 and 180 DIV. Values represent mean +/− standard error of mean (SEM) from *n* = 3 hMBOs per group. Three sections were analyzed from each hMBO. The results were analyzed using two-way ANOVA [*SNCA* copy number: F (1, 8) = 20.13, *p* = 0.0020; Time: F (1, 8) = 12.65, *p* = 0.0074); *SNCA* copy number × time: F (1, 8) = 0.6670, *p* = 0.4377)] followed by the Tukey post hoc multiple comparisons test. * *p* < 0.05.

To further investigate this, we immunostained the tissue sections with an antibody against cleaved caspase 3 (c-Casp3), a marker of cellular apoptosis (Figure 6A [120 DIV] and B [180 DIV]). The presence of apoptotic areas has been previously reported in the brain organoid model [49]. In agreement, we noted a basal level of apoptosis in the healthy hMBOs, both at 120 and 180 DIV (Figure 6A,B). However, the level of c-Casp3 immunostaining was higher in the fPD-hMBOs at both time points (Figure 6A,B). Image analysis confirmed that there is indeed a significant increase in c-Casp3 immunostaining in the fPD-hMBOs compared with the healthy-hMBOs at 180 DIV (Figure 6C).

## 4. Discussion

In this study, we aimed to model key histopathological features of PD, including the formation of LB-like αSyn inclusions and loss of DA neurons in the hMBOs without any genetic alteration. Recently, multiple groups attempted modeling PD-like pathology in hMBOs carrying different PD-associated mutations. Smits et al. used midbrain-specific organoids generated from PD patients carrying the LRRK2-G2019S mutation and demonstrated a reduction in the function and number of DA neurons in LRRK2-G2019S compared to the control organoids [29]. Likewise, Kim et al. generated isogenic 3D midbrain organoids with or without a PD-associated LRRK2 G2019S mutation. They reported an increase in the levels of phosphorylated αSyn (pS129) and deterioration of DA neurons, as well as decreased neurite length, indicating that the organoid model system did recapitulate some aspects of PD pathology [50]. On the other hand, Nickels et al., 2020, used PD-inducing toxins and observed alterations at the cellular level, such as neurotoxicity in hMBOs [25]. Nevertheless, these studies did not report LB morphogenesis, a cardinal histopathological characteristic of PD pathophysiology. It is crucial to model this hallmark feature of PD pathology for a comprehensive understanding of disease mechanisms and the development of therapeutic interventions. Recently, Jo et al., 2021, have been able to generate LB-like inclusions in hMBOs by knocking out glucocerebrosidase (GBA1) and overexpressing αSyn [51]. In the current study, we investigated the spontaneous appearance of LB-like pathology in hMBOs derived from PD patient-iPSCs without any genetic manipulation. Duplications and triplications of the *SNCA* gene have been found in familial forms of PD [52,53,54]. While duplications are associated with a phenotype resembling sporadic PD, triplications are associated with a more aggressive phenotype, including earlier age of disease onset and more severe motor symptoms [55]. In this study, we generated hMBOs from PD patient hiPSCs carrying *SNCA* triplication. These fPD-hMBOs displayed a gradual increase in pathological αSyn, which accumulated into LB-like inclusions and the associated neurodegenerative alterations typical of PD, including apoptosis and the loss of DA neurons. Although we observed spontaneous appearances of LB-like αSyn deposits, we were unable to identify Lewy neurites.

In PD and other synucleinopathies, more than 90% of αSyn in the LBs are phosphorylated [56]. The abundance of pS129-positive αSyn inclusions significantly correlates with neurodegeneration and clinical phenotypes in PD; therefore, phosphorylation at serine 129 is used to distinguish normal αSyn from abnormal αSyn, particularly αSyn in proteinaceous inclusions [57]. In this study, we noted increased pS129-positive αSyn immunoreactivity over time in the fPD-hMBOs. However, we were unable to detect pS129-positive αSyn immunoreactivity and insoluble αSyn in the hMBO homogenates using biochemical assays. This is most likely due to a low abundance of cells containing αSyn deposits (Figure 3B) and the small size of the organoids compared with the human brain. Furthermore, LB-like αSyn deposits were not consistently observed in all the batches of fPD-hMBOs. Nevertheless, we have noted a consistent and progressive increase in pS129 immunostaining over time, a key feature of PD pathology (Figure 3). It is possible to culture brain organoids for 300–600 days [22,58]. Long-time hMBO culture may help generate more extensive LB-like pathology and help us estimate the levels of insoluble and phosphorylated αSyn via biochemical techniques. In addition, multiple PD-associated mutations can be introduced to generate a more aggressive phenotype in a shorter time frame [51].

Classic, fully mature LBs are spherical cytoplasmic inclusions with smooth edges, characterized by hyaline eosinophilic cores, concentric lamellar bands, and peripheral halos, as well as immunoreactivity for αSyn and pS129-αSyn [44,45,59]. Histopathological studies suggested that LB formation may involve different stages [44]. We compared the αSyn deposits observed in the fPD-hMBOs with the LBs present in LBD patient brain sections. αSyn inclusions in fPD-hMBOs were remarkably similar to the brain resident LBs with respect to morphology, intracellular localization and marker composition (Figure 4). However, the core and pS129-positive lamellar band were not apparent. This internal organization may require further maturation. On the other hand, we noted pale body (PB)-like immunostaining in the hMBOs (Figure 3B and Figure 4B). PBs are present in the early stages of LB formation [45]. These are more intracytoplasmic and irregular in shape, with some glassy areas intensely immunolabeled with anti-pS129 [44]. p62 is another well-established marker of LBs [44,46]. The p62/sequestosome 1 is a selective cargo receptor for autophagy in the degradation of misfolded proteins. The pink1/parkin mediated mitophagy pathway is also dependent on the p62/sequestosome [60]. Importantly, p62-induced autophagy failure significantly accelerates misfolded protein aggregation [61]. p62 immunostaining in fPD-hMBOs and human LBD brains revealed similar patterns of perikaryal p62 accumulation. Furthermore, we also detected small intranuclear p62 inclusions in fDP-hMBOs (Figure 4C), similar to the findings of Kuusisto et al. from LBD brains [44].

Finally, to investigate the PD-like degeneration of DA neurons in hMBOs, we immunostained the section for TH and cleaved caspase-3 (c-Casp3) (Figure 5 and Figure 6). TH immunostaining was significantly decreased in fPD-hMBOs (Figure 5) and they displayed elevated levels of c-Casp3 levels (Figure 6), indicating increased apoptosis, as observed in the PD patients’ brains [62].

One of the limitations of our study is that the age in which iPSCs were collected in the fPD patient is younger than the healthy subject. Also, the reprogramming methods for these two lines were not identical. However, we were unable to find any evidence from the literature that donors’ age or reprogramming method might influence αSyn pathology or any neurodegenerative disease pathology per se in iPSC-derived model systems. In conclusion, our work provides an hMBO-based model of PD, which recapitulates the key pathological features of the disease, including the accumulation of pS129-positive αSyn over time, the formation of LB-like inclusions and DA neuron loss. We believe this model will complement the existing experimental models of PD and significantly contribute to biomarkers and therapeutic development.

## Data Availability

Data and materials will be provided upon request to the corresponding author.

## Supplementary Materials

The following supporting information can be downloaded at: https://www.mdpi.com/article/10.3390/cells12040625/s1, Figure S1: characterization of the hiPSCs; Table S1: antibodies used for immunofluorescence.
