# Supplementary material for: Lewy Body-like Pathology and Loss of Dopaminergic Neurons in Midbrain Organoids Derived from Familial Parkinson’s Disease Patient
Source: Cells. 2023 Feb 15;12(4):625. doi: 10.3390/cells12040625 (PMC9954141; doi:10.3390/cells12040625)
Supplement: Supplementary file 1 [file cells-12-00625-s001.zip › cells-2095456-supplementary.pdf]

Supplementary Information

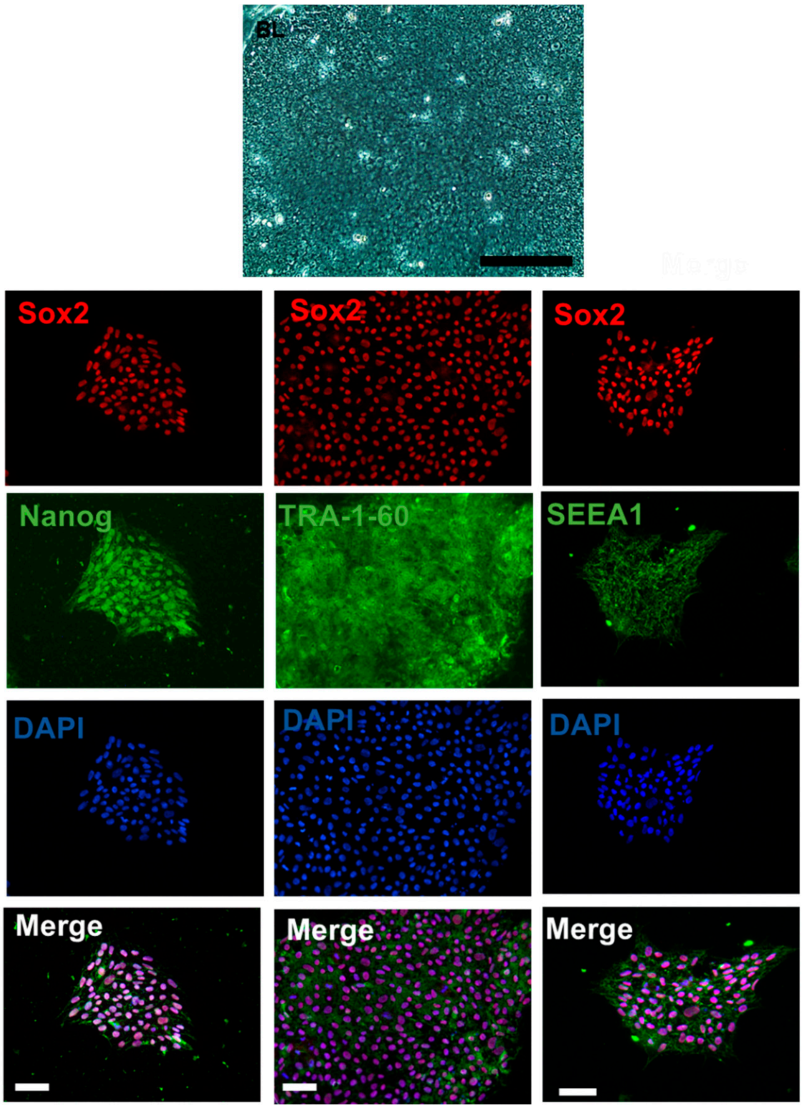

Figure S1. Characterization of the hiPSCs

Morphological and immunofluorescence based characterization of the healthy hiPSCs. BF, representative Brightfield image of hiPSCs. Characterization of healthy hiPSCs using pluripotency markers (Sox2, Nanog, SSEA1, TRA-1-60). Scale bar 50 μm.

Table S1. Antibodies used for immunofluorescence

Antibody	Host species	Company (catalog number)	Dilution IF and IHC
GFAP	Mouse	Thermo Fisher scientific	1:500
GFAP	Rabbit	Abcam (ab7260)	1:500
αSyn 506	Mouse	BD Bioscience (610787)	1:200
c-Casp3	Rabbit	Abcam (ab230)	1:500
En-1	Rabbit	Thermo Fisher scientific (PA5-14149)	1:200
LMX-1A	Rabbit	Abcam (ab139726)	1:200
MAP2	Mouse	Thermo Fisher scientific (BDB556320)	1:500
MAP2	Rabbit	Abcam	1:500
NURR1	Mouse	Thermo Fisher scientific (MA1-195)	1:200
OTX-2	Rabbit	Abcam (ab1990)	1:200
pS129	Rabbit	Abcam (ab51253)	1:200
SOX2	Rabbit	Abcam (ab7959)	1:500
TH	Rabbit	Millipore Sigma (AB152)	1:200
TUJ1	Mouse	Biolegend (825702)	1:500
P62	Mouse	Abcam (ab109012)	1:200
